# Food Security and the COVID-19 Crisis from a Consumer Buying Behaviour Perspective—The Case of Bangladesh

**DOI:** 10.3390/foods10123073

**Published:** 2021-12-10

**Authors:** Mohammad Fazle Rabbi, Judit Oláh, József Popp, Domicián Máté, Sándor Kovács

**Affiliations:** 1 Károly Ihrig Doctoral School of Management and Business, University of Debrecen, 4032 Debrecen, Hungary; drrabbikhan@gmail.com; 2 Faculty of Economics and Business, University of Debrecen, 4032 Debrecen, Hungary; kovacs.sandor@econ.unideb.hu; 3 College of Business and Economics, University of Johannesburg, Johannesburg 2006, South Africa; popp.jozsef@uni-neumann.hu (J.P.); mate.domician@eng.unideb.hu (D.M.); 4 Hungarian National Bank–Research Center, John von Neumann University, 6000 Kecskemét, Hungary; 5 Faculty of Engineering, University of Debrecen, 4032 Debrecen, Hungary

**Keywords:** food security, COVID-19, food supply chain management, consumer behaviour

## Abstract

Since COVID-19 was confirmed in Bangladesh in March 2020, the government have enacted stringent measures to prevent the spread of the coronavirus, which has had a significant impact on people’s lives. Food consumption habits of consumers have shifted as a result of declining grocery shopping frequency, negative income shock, and food prices shooting up. This paper aims to explore Bangladeshi consumers’ buying behaviour in association with the stress generated from a food supply shortage during the COVID-19 pandemic and the post-outbreak perception of the food industry, using a dataset with 540 online samples collected between July and August 2021. A two-stage cluster sampling method and self-administrated questionnaire techniques were adopted for collecting the data during the third wave of COVID-19. Using partial least squares path modelling (PLS-PM) and multivariate multiple ordered logit regression (MVORD) to reveal the pertinent structure between all the blocks, this study provides two key findings. First, a higher intensity of COVID-19 impact translates into higher food stress associated with income reduction and higher food prices. Second, food stress directly affects consumer buying and consumption behaviour. We strongly recommend connecting consumers with local producers and collective use of shared warehouses through institutions, policies, and reforms to prevent disruption in the food supply chain and to keep food prices stable. Additionally, food producers, distributors, stakeholders, and policy planners should strengthen the food supply chain to stabilize food security.

## 1. Introduction

The coronavirus outbreak rapidly surged worldwide and turned into a global pandemic [1]. Almost all low, middle, and high income countries, including Bangladesh, implemented non-therapeutic preventive measures such as travel restrictions, remote office operations, and most notably, lockdown and social distancing to flatten the curve of virus infection [2]. The COVID-19 pandemic and lockdown have affected the most vulnerable groups and the economy as a whole in three main ways, including direct output impacts, instability of the supply chain and financial markets, and multiple economic implications [3]. The Bangladeshi agricultural sector is the primary source of the food supply across the country, but agriculture and the food sector in Bangladesh is expected to lose approximately USD 625 million as a result of the pandemic [4]. COVID-19 has wreaked havoc on the world’s agricultural systems, from production to distribution, consumption, and trade. Since the COVID-19 onslaught in Bangladesh, farmers have lost an estimated625 million United States dollars (USD) [5,6]. The Bangladesh poultry industry lost an estimated USD 135 million, and the dairy industry lost USD 6.7 million a day due to the wastage of 12–15 million litres of milk. Large amounts of eggs, milk, fruits, and vegetables were thrown away due to a lack of suitable storage facilities [6]. Food loss during the pandemic was also exacerbated by an increase in transportation costs, non-availability of labour, and inadequate storage facilities.

The United Nations (UN) and its constituent entities are primarily focused on the food supply chain (FSC) in this global disaster. As COVID-19 becomes more severe, the FSC interruptions will become a crisis, outpacing the communicable disease itself [7]. Food demand and consumption are both adversely affected. Prior to COVID-19, developing and impoverished countries such as Bangladesh were already struggling with unstable FSCs, interrupting even minor food security risks [8]. Moreover, travel restrictions have hampered farmers’ access to agricultural inputs and prevented producers from accessing markets. The number of vehicles on the road has dropped drastically. As a result, food transportation prices have increased [9]. Global food production and consumption gained tremendous attention during the pandemic because of the severe issues created in meeting global food demand [10,11,12]. In a highly densely populated country such as Bangladesh, the pandemic greatly impacted the food and manufacturing sectors. Moreover, it becomes apparent that negative agri-food trade moves towards a state of higher vulnerability, especially when certain trade restrictions are applied [13]. Farmers face severe financial difficulties as a result of decreasing demand for their products, supply disruptions, and being compelled to sell below cost. Many people rely on credit, and their credit lines are being shut off. A continuation of the COVID-19 situation will have a negative impact on food production in general [6].

COVID-19 eventually had a massive impact on food production and supply, ultimately disrupting it. Dabo Guan et al. observed that the COVID-19 problem threw supply chains into disarray [14]. Demand and supply ripples were seen, as well as chaos and resonance effects that spread across the world. Consequently, supply shortages are visible in retail shops in Bangladesh, and stress in consumer behaviour is apparent. Almost all parties involved in food production and distribution are affected, resulting in negative effects on food quality, freshness, and food safety, and impeding consumer access and affordability [13]. Poor households are consuming cheaper and less nutritious foods in order to manage rising food prices, accompanied by declining purchasing power, often losing the quality of their diet. According to a survey carried out by the Bangladesh National Nutrition Council (BNNC), 75% of respondents reported that they did not have enough food at home, while 91% stated that they did not have enough money to buy food [4]. Moreover, daily wage earners were hit by a negative income shock as a result of the lockdown.

In addition, rising unemployment and widespread job losses in both the formal and informal sectors, low wages, higher food prices, and scarce food in local stores have led to declining purchasing power. Prices of essential foods have continued to increase in several countries during COVID-19, when people have very little cash in hand [15,16]. In the presence of increasing stringency measures, the relative backwardness which stops economic activity and a reduced socioeconomic position leads to extreme poverty, hunger, criminality, and malnutrition. The pandemic is the greatest health crisis of our time, and it is a global humanitarian catastrophe which has put millions of people across the globe at risk of food security difficulties and poor nutrition. Millions of people were already starving and malnourished before the epidemic hit, and global food shortages will occur unless urgent action is taken. According to the Global Economic Prospects (GEP) report, the pandemic could have pushed between 119 and 124 million people into extreme poverty by 2021 [17]. The epidemic is also likely to exacerbate financial and gender inequality because of its disproportionate impact on women, children, and unskilled and informal workers, as well as its detrimental consequences on education, health, and living conditions [18]. During pandemics, food and nutrition security became a major concern, and food supply disruptions could lead to a rapid food crisis in the near future [19]. Additionally, diet changes as food supply and access improve through this crisis.

The global COVID-19 pandemic exposes significant vulnerabilities, disparities, and system-wide threats in food supply systems, which should promote strategies toward greater food supply chain sustainability and resilience. Khan et al. investigated the idea that poorly designed food supply chains (FSC) are viewed as the root cause of food insecurity and widespread malnutrition [20]. Cariappa et al. identified that the disruptive shocks in pricing and food value chains cause distortions in agricultural commodity distribution and consumption, resulting in significant effects on food price and consumer behaviour [21]. Laborde et al. argued that the pandemic’s most significant impact on food security is a decrease in income which jeopardizes food access, unless social safety-net programs are well matched to the challenge [22].

However, predictions indicate a near doubling in food insecurity globally due to increasing unemployment, job losses, food supply chain disturbances, and other responses to the pandemic, such as disturbances to social welfare systems in Bangladesh. Several research studies have covered COVID-19 in its disruption of the entire production and distribution process, the food supply chain, food quality, and food security [20,22,23,24]. However, during the COVID-19 lockdown, how food stress and external factors have changed the Bangladeshi consumer’s buying behaviour and food security status has not been explored, and this question forms the research gap. This study aimed to explore consumer buying behaviour in connection with the stress caused by the lack of food supply during the disruption of COVID-19 and the perception of the food industry after the outbreak. This research intended to answer some specific queries, as justified by the relevant literature studies: first, what are the factors that affect food production and the food supply chain during COVID-19; second, what are the relationships between the proposed variables (i.e., food security measures and nutrition changes during COVID-19); third, how do consumers behave in order to cope with the stress generated from a food supply shortage during a crisis as COVID-19; and finally, what might be the perceptions of the post-outbreak situation?

## 2. Literature Review

### 2.1. Theoretical Background and Consumer Behaviour during COVID-19

As COVID-19 spreads across the world, concerns of a major global recession are rising. COVID-19 is both an emergency for public health and safety and an experiment in downsizing the consumer market in real-time. Consumer habits disrupt four major contexts. First, changes in the social context include different life events such as workplace, community, neighbours, and friends. Second, context is altered by new innovative technologies. Third, consumption habits are impacted by rules and regulations. Finally, unanticipated natural catastrophes such as earthquakes, floods, and global pandemics—including the COVID-19 outbreak—all affect consumers’ consumption behaviour. Several civil wars and global wars (the Cold War, World Wars I and II), regional conflicts, the great recession, and great depressions, have radically interrupted consumers’ consumption behaviour and the food production and supply chain.

Several previous research studies have demonstrated the necessity of looking at consumer behaviour in times of global crisis [25,26,27]. Sheth [28] identified eight instruments of the COVID-19 pandemic related to consumption and consumer behaviour, including stockpiling, improvisation, holed-up demand, adopting new technologies, the shop comes into the home, blurring of work-life boundary lines, gatherings with friends and family, and the discovery of skills and abilities. The theoretical framework of this scientific research is based on integrating elements of the theory of food security [29,30,31] and the theory of reasoned action (TRA) [32] that explain one’s intention to perform a certain consumer behaviour. Nevertheless, the formally accepted concept of food security was developed after the World Food Summit of 1996. Food security occurs when all individuals have physical and economic access at all times to adequate, secure, and nutritious food that satisfies their nutritional requirements, and they have food choices for a healthy and balanced life [29,31,33]. However, in this current study, the authors developed a conceptual research framework based on the theoretical background of the four pillars of food security, including availability and accessibility. Here we considered food quality and safety under food utilization and food price under food stability. We considered one variable of consumer behaviour from the theory of reasoned action (TRA).

**Figure 1:**
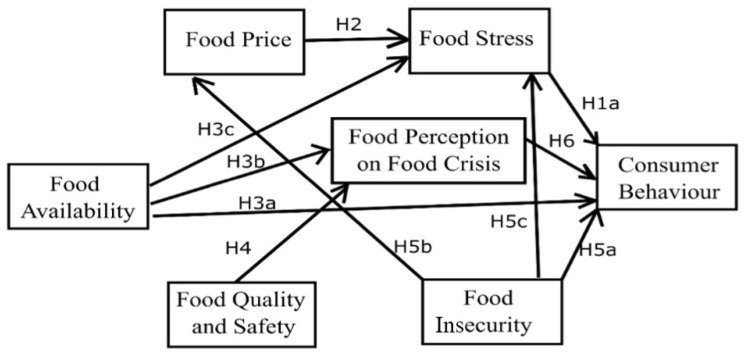
Proposed Conceptual Framework. Notes: H denotes hypothesis; conceptualization of the network is based on the theory of reasoned action (TRA). Source: Authors’ own compilation.

Moreover, the authors extended two variables with food stress and future perception of food crises with consumer behaviour during COVID-19. Several previous studies have measured food security by using three pillars of food security [33,34,35]. Here, we propose a conceptual research framework and hypotheses (denoted by H) in Figure 1, where consumer behaviour serves as the dependent variable, and food stress, food price, food availability, food insecurity, food quality and safety, and future perception on the food crisis serve as the independent variables used to detect the effects among the variables and the model fit. Moreover, the conceptual research model can support estimates of the current consumer’s behaviour on food security in a post-pandemic context.

### 2.2. Food Stress

Government declarations of lockdowns have led to panic purchases of food in most nations, which leads to a scarcity of goods and services [36]. International supply chains are in disorder, and operators are looking for local sources of fictional materials to maintain industrial production. Even consumers are stockpiling non-perishable food for future consumption [37]. Social distancing is a form of social isolation that has a negative impact on the well-being of consumers [38,39]. The consequences of COVID-19 social isolation generate consumer disorders such as depression, anxiety, and stress [40,41]. Additionally, food is not available in the local store, and the price of staple food has increased by 300%. Therefore, consumers are concerned about food running out. Disruption of the food supply chain, unavailable food, sold-out food, price increases, and joblessness during the pandemic all contribute to consumer food stress [42]. Thus, food stress has a significant influence on consumer behaviour during the pandemic. Thus, we demonstrate that:

**Hypothesis** **1a** **(H1a).** *Food stress has a direct effect on consumer behaviour*.

**Hypothesis** **1b** **(H1b).** *Food stress has a direct effect on future perceptions of the food crisis*.

### 2.3. Food Price

During COVID-19, food prices soared, wreaking havoc on millions of families worldwide, leading to food production and supply shortfalls, and contributing to the unconscionably high levels of chronic hunger around the world. Panic stockpiling also impacted consumers’ price sensitivity. Previous studies (e.g., Huang et al. [43]; Pantano et al. [44]) have examined the predominance of determining consumer price elasticity. Thus, it is not surprising that many suppliers have raised prices by up to 300% for certain product segments during emergencies. According to the Transparency International report of 2019 [45], Bangladesh is the 14th most corrupt country globally, where wholesalers and retailers have already increased prices by up to 400%. Persistent increases in prices (inflation) have been a common sight during the national crisis in Bangladesh. Food price is an important factor that significantly influences consumers’ behaviour during the COVID-19 outbreak. Moreover, increasing food prices are a cause of stress. Thus, this leads to the following hypothesis:

**Hypothesis** **2** **(H2).** *Food price has a direct effect on food stress*.

### 2.4. Food Availability

The perceived lack of availability of products can significantly impact consumer preferences [46]. Scarcity increases the importance of unavailable goods [47]. Consumer purchase behaviour and stress during the pandemic also depend on food available in the retail stores. According to McKinsey & Company, stockpiling and product unavailability are disrupting consumers [48]. However, more recent studies have reported that food supply will be severely affected and that the number of hungry people will double if governments intervene.

Moreover, pandemic-induced lockdowns could double the number of hungry people [49]. Countries must keep their food supply chains functioning to minimize potential food shortages. Following this, the FAO proposes unique measures such as extending emergency food relief programs and offering urgent support to smallholders’ agricultural production by enhancing electronic commerce.

Similarly, it recommends concentrating on the key logistical blockages (e.g., disrupted food transportation through sticks and perishable commodities such as fish, vegetables, and fruits), reviewing tax and trade policies to keep the supply chain functioning, and introducing monetary incentives in the event of food prices soaring [50,51]. Therefore, food availability has a significant relationship with consumer behaviour. Based on the literature, these estimates are tested in this research in the following assumptions:

**Hypothesis** **3a** **(H3a).** *Food availability has a direct effect on consumer behaviour*.

**Hypothesis** **3b** **(H3b).** *Food availability has a direct effect on future perceptions of the food crisis*.

**Hypothesis** **3c** **(H3c).** *Food availability has a direct effect on food stress*.

### 2.5. Food Quality and Safety

Previous coronavirus-related outbreaks, especially MERS-coronavirus and SARS-coronavirus, have shown that food is not a transmission pathway for these specific viruses [52]. Transmission is possible if the affected person touches food and, shortly after that, another person picks it up and touches the eyes or mucous membranes of the mouth or throat [24]. To reduce any potential risk of touching food infected with the coronavirus, extensive hand-washing or sanitizing should follow the handling of food packages [53]. However, it is mainly the precautions relating to food handling and planning processes recommended by the WHO which prevent cross-contamination between cooked and uncooked foods [54]. Food quality and safety have already decreased in Bangladesh, and in fact, some unscrupulous businessmen have earnt more money by mixing adulterated food during the outbreak. Therefore, consumers are under great stress, and their purchase behaviour is changing quickly, and food quality and safety have a significant influence on consumer behaviour and future expectations regarding food during any pandemic. Thus, our assumption is that:

**Hypothesis** **4** **(H4).** *Food quality and safety have a direct effect on future perceptions of the food crisis*.

### 2.6. Food Insecurity

The fastest growing concern worldwide is food insecurity, and it is estimated that over 1 billion people have food consumption which is insufficient to meet the energy needs of the diet (chronic hunger), and twice that number are suffering from micronutrient deficiencies (hidden hunger) [55]. Over 2 billion people are suffering from hidden hunger, and approximately 800 million people are chronically starving [56]. According to FoodForward, “food insecurity refers to a lack of access to enough good, healthy, and culturally appropriate food” [57]. The COVID-19 epidemic has become a presumed result of the intercontinental food crisis, posing an imminent threat to the food and nutrition environment, which is expected to be extremely dangerous among the most vulnerable populations [56]. According to the Global Network Against Food Crisis 2020, the COVID-19 pandemic could lead to acute food insecurity for 265 million people who need urgent food and survival assistance [58]. The imbalance of food security determinants is caused by decreases or deficiencies in aggregate food availability, accessibility, and utilization at local, regional, national, or global levels [59]. Instead of focusing on food availability at the micro-level, more attention is needed at the macro level and to create the resilience of food availability at the household level access to the income streams must be ensured.

Additionally, by enacting food security policies, food consumption capacity can be enhanced [60]. Global food security alerts have emerged. Food systems incorporate all the various stages of food processing from farm to the ultimate consumer (e.g., methods of production, delivery and preparation, consumption, and eventually discharge) and all the major components concerned (e.g., transportation, farming products, ecosystems, farmers, manufacturers, suppliers, and organizations) [61]. Food security is also an essential aspect that greatly affects the behaviour and stress of customers. Therefore, we hypothesize:

**Hypothesis** **5a** **(H5a).** *Food insecurity has a moderate effect on consumer behaviour*.

**Hypothesis** **5b** **(H5b).** *Food insecurity has a direct effect on food prices*.

**Hypothesis** **5c** **(H5c).** *Food insecurity has a direct effect on food stress*.

### 2.7. Future Perceptions of the Food Crisis

There are currently 820 million people worldwide without adequate food, and COVID-19 is expected to drive 130 million more to the verge of starvation, more than doubling the figure to 265 million by the end of the year. The International Food Policy Research Institute (IFPRI) claimed that developing countries are not the only ones facing a poverty and hunger crisis, but also over 140 million people could fall into extreme poverty globally [62]. Future perceptions of the food crisis will also increase in Bangladesh. Consumers in Bangladesh are concerned about the whole food supply chain process, food prices, food security, food quality, and the food crisis. Consumer behaviour has a negative perception of the food crisis. Thus, we can propose the following research hypothesis:

**Hypothesis** **6** **(H6):** *Future perceptions on food crises have a direct effect on consumer behaviour*.

## 3. Materials and Methods

This research was carried out on quantitative models, and a questionnaire was used to survey a research instrument during the COVID-19 pandemic to study consumer behaviour in Bangladesh. The study area of Bangladesh was a deliberate choice. After India, Bangladesh is the second most affected country by COVID in South Asia. A combination of both primary and secondary data was used to conclude the research.

### 3.1. Questionnaire Development and Instrument

A self-administered questionnaire was developed to collect the survey data used to measure the eight variables on consumer buying behaviour and food security by research scholars, PhD research fellows, academicians, and food supply chain experts. We used focused group discussion (FGD) and news content analysis methods to prepare the final items for the questionnaire as an explanatory study. Content analysis is a research methodology widely applied to phenomena such as propaganda, literature and newspapers, psychotherapy transcripts, and TV programming [63]. We used content analysis and the FGD method during the COVID-19 pandemic, and there was no empirical study on consumers’ food purchasing behaviour. Instead of conforming to known dimensions of food security, first, we analysed content from newspapers, world reports, organizations’ reports and television news, where news was related to the food crisis, consumer stress, food supply-chain management problems, food storage and shortage problems, export and import problems, retail shop problems, and consumer behaviour during COVID-19. After content analysis, we revealed four new important variables and items. The authors adapted food quality and safety variables under food utilization and food price under food stability from established pillars of food security [64,65] and consumer behaviour from TRA [32]. We had to modify some new items under consumer behaviour (CB), food stress (FS), food availability (FA), food price (FP), and food quality and safety (FQS) based on content analysis to achieve the research objectives and to identify items relevant to food issues with COVID-19. Then, an online video call conference was arranged for focus group discussion (FGD) with PhD research fellows, academicians, and three supply chain experts in the food industry in Bangladesh. After deliberation, we picked two new food stress and food perception variables on food crisis variables and items from four variables consistent with the COVID-19 pandemic. In addition, we also obtained new items under CB, FA, FP, FQS, and FS related to the food supply chain, food shortages, and consumer perception of food during COVID-19.

Finally, seven variables (consumer behaviour, food stress, food price, food availability, food quality and safety, food insecurity, future perceptions on food crisis) were selected, involving 18 items measured on an ordinal scale that were used to represent non-mathematical ideas such as frequency, satisfaction, enjoyment, and degree of stress [66]. Thus, an ordinal scale was used to determine stress on consumer buying behaviour [43]. To design the measurement-related question, a 5-point Likert scale was applied to independent and dependent variables, where 1 = strongly disagree, 2 = disagree, 3 = neutral, 4 = agree, and 5 = strongly agree. Before finalizing the questionnaire and guaranteeing the correct conceptual framework, the questionnaire was pre-tested by 2 research scholars, 3 food industry experts, and 20 random consumers by sending questionnaires via email and social media (Facebook). After pre-testing, some items were omitted because of ambiguous, inconsistent, and improper responses. The final version of the questionnaire was designed to provide a respondent’s perspective with a more distinguishable pattern. The collected data were analysed and presented to illustrate the linkages between the proposed variables.

### 3.2. Participants and Procedure

A simple two-staged cluster sampling method was adopted, following the instructions of Malhotra and Dash [67], and a self-administrated questionnaire was distributed to Bangladeshi people randomly by social media (Facebook). Face-to-face questionnaires could not be conducted because of social distancing and lockdown situations in the whole of Bangladesh. We used Google forms for an online survey with a pretested and well-structured questionnaire (see supplementary file) which was conducted during the third wave of the pandemic between 1 July 2021 and 5 August 2021 and translated the questionnaire into the Bengali language in order to obtain appropriate comments from respondents. All respondents were involved in several professions employed in various organizations and lived in different cities in Bangladesh. The cluster sampling technique was used for selecting respondents from Bangladesh during the pandemics. A nationwide locked down was imposed from 1 to 13 July [68] and reimposed the from 23 July to 5 August 2021 [69]. For the Muslim people’s biggest Eid-ul-Adha festival celebrations, all the restrictions were relaxed from 14 to 22 July 2021 [70]. Six Facebook groups were involved which were connected to scientific research: Research and Development Professionals in Bangladesh, Stipendium Hungaricum Scholarship for Bangladesh, Research Help Bangladesh, Research Support Group—Bangladesh, Bangladeshi Young Researchers, Bangladesh Research and Innovation Society. In the first stage, three groups were randomly selected, and in the second stage, respondents were selected according to a simple random sampling within each cluster.

Finally, out of 900 respondents, 610 returned the completed questionnaires as samples for analysis. A total of 70 questionnaires were dropped due to respondents’ lack of skill, unconscious approach, and absence of basic standards. Finally, 540 samples were selected for this research, consisting of 73% males and 27% females. In terms of age group, the majority were 20–30 (67%) years of age; those aged 31–40 accounted for 23%, and those aged 41–50 for 8%; those above 51 constituted 2% of the respondents. In terms of jobs, the majority of respondents were private job holders (25%), government employees (17%), students (25%), homemakers (4%), unemployed (10%), and working in agriculture (5%), business (2%), and other professions (4%). Furthermore, 15.9% of respondents had a monthly income of USD 126–250, 13% received USD 251–375, 13% received USD 376–500, and 12.4% USD 500.

Online surveys limit data collection from illiterate people and those without access to technology. As a result, all of our respondents were literate, with approximately 75% having a graduate degree. Most of the households had 4 to 5 members.

### 3.3. Data and Pre-Processing

The data consisted of 540 responses and were structured into seven blocks (consumer behaviour, food stress, food price, food availability, food quality and safety, food insecurity, future perceptions of food crises) involving 18 items measured on an ordinal scale. The sample size can be considered sufficient for further analysis regarding partial least squares, as a minimum of 200 individuals is required [71,72,73]. There were no missing data, nor registration errors, and multicollinearity levels were acceptable, as the variance inflation factor (VIF) values were around 3.0 or less [74]. Collinearity diagnostics were calculated with the items of consumer behaviour as the dependent variable, and the other items were the independent variables, using the “olsrr” package in R software [75].

### 3.4. Applied Methods

#### 3.4.1. Multivariate Multiple Ordinal Logit Regression

The primary aim of the study was to develop a path model. To form more comprehensive blocks within the model, we applied a preliminary multivariate multiple ordinal logit regression (MVORD) to reveal the relevant structure between the blocks and test the hypotheses. The “mvord” package was used to carry out the calculations [75]. This regression model extends the general linear model to ordinal categorical data with several outcome variables. The so-called logit link function, i.e., the log of odds (the quotient of the cumulative probabilities for the *r_ij_*th category), was used in the estimation, resulting in a linear model of the parameters as follows [76]:

(1) $$ln\left(\frac{P(Y_{ij}>r_{ij}}{P(Y_{ij}\leq r_{ij}}\right)=\overset{}{\underset{v}{\sum }}x_{ijv}\cdot \beta_{jv}-\theta_{j,r_{ij}},$$

where $\beta_{jv}\;$ is the parameter of the *v*th covariate for the *j*th outcome, $\theta_{j,r_{ij}}\;$ is the threshold variable of the *i*th individual and *j*th outcome for *r_ij_*th category, *Y_ij_* is the value of the *j*th outcome for the *i*th individual, and $x_{ijv}$ is the value of the *v*th covariate for the *j*th outcome variable for the *i*th individual. The threshold values are not of much interest. Their role is similar to that of intercepts in linear regression. Let ${\tilde{x}}_{ijv}$ denote a 1 unit increase in the *v*th covariate of the *j*th outcome for the *i*th individual and hold all the other covariates constant. The relationship between the odds before and after the change can be modelled as follows [76]:

(2) $$exp\left[\beta_{jv}\right]=\left(\frac{P(Y_{ij}>r_{ij}|{\tilde{x}}_{ijv}}{P(Y_{ij}\leq r_{ij}|{\tilde{x}}_{ijv}}\right)/\left(\frac{P(Y_{ij}>r_{ij}|x_{ijv}}{P(Y_{ij}\leq r_{ij}|x_{ijv}}\right),$$

where $exp\left[\beta_{jv}\right]$ represents the relative risk of the *v*th covariate on the *j*th outcome.

Positive relative risk values mean that when the value of the given covariate increases, the odds of larger against lower values of the *j*th outcome increase. The negative parameter means the opposite: the odds of larger values decrease.

#### 3.4.2. Path Modelling

The final path model was fitted by the partial least squares path model (PLS-PM) [77,78,79]. The construction of the model was based on the structure identified by the MVORD analysis. Researchers utilized a considerable piece of PLS-PM for the utilization of the MVORD analysis. PLS-PM can estimate complex path models with many variables and objects, as compared with PLS-SEM. Only two items were left out of the model for conceptual reasons due to the low factor loadings (cb1 and pfpc3). However, the formative [80,81] and the reflective model LVs [82] could be supported; we applied the reflective method of modelling in our research. The overall model fit was measured by a global criterion of goodness-of-fit (GoF) [83], and for the validation we employed bootstrapping using 500 samples according to Chin [79]. The GoF of 0.10, 0.25, 0.36 can be considered an adequate, moderate, and good global fit [84]. Dillon Goldstein’s rho index tested the composite reliability of the blocks. The dimensions identified should have reliability above the recommended 0.7, and factor loadings should exceed 0.6–0.7 [73]. For assessing the quality of the structural model, R^2^ values were calculated. The values of 0.02. 0.15, 0.35 are considered as small, medium, or large effects, according to Cohen [85]. In order to check the discriminant validity of the model, the Fornell and Larckner criterion was used [86]. That is, the average variance extracted (AVE) of a latent variable (LV) should be higher than the amount of variance explained in other LVs by this latent construct. The model fit and calculations were performed by the ‘‘plspm” package in R software [72,75].

The PLS approach has several advantages in comparison with the classical econometric regression models. First of all, PLS minimizes the complications of measurement error in the model [87] and addresses the difference between the direct and indirect effects with a mediator variable. Second, the technique can detect all the paths between the variables [88] and can handle correlated independents (multicollinearity) between the manifest variables and the system of structured mediation effects with multiple manifest variables [89]. In the classical regression models, which use the ordinary least square regression (OLS) or weighted least squares (WLS), multicollinearity often causes a problem. As a solution to this issue, highly correlated variables could be removed from the model, which can cause biases and a loss of information. Król [90] stated that especially in the case of a large number of categorical predictors and multicollinearity, as in our case, the PLS approach might be a better alternative to the classical methods. PLS modelling also allows a non-parametric approach for testing parameters when normality is violated [78]. The above-mentioned reasons guided us to prefer PLS latent variable path modelling.

## 4. Results and Discussion

The following 7 question blocks were examined: consumer behaviour (3 items), food stress (2 items), food price (2 items), food availability (2 items), food quality and safety (3 items), food insecurity (2 items), and future perceptions of food crises (4 items) (Table 1). We kept only those items whose loading was larger than 0.7. Hence, from the final path model, FPFC3, FQS2, and CB3 were left out. After excluding these factors, several loadings were improved for the whole model fit. Consumer behaviour (CB) is best described by “food hoarding” (0.919) and food stress by the “concern about running out of food” (0.927). FQS basically depends on “low quality and adulterated foods” (0.947), and future perceptions of food crises are strongly related to the fear of “a big food crisis after the COVID-19 pandemic” (0.904). Moreover, the reliability of the scale items was established through the score of the Cronbach’s alpha value. Studying the descriptive statistics, we could obtain the highest averages with lower standard deviation in the case of CB1 and FP1, while the lowest average scores were given to safety issues (FQS2) and the satisfaction of the daily nutrition supplies (FI2).

At the first stage of the analysis, a preliminary ordered logit analysis was performed on all the items to reveal the relevant blocks of items and reduce the dataset to achieve better composite reliability (Table 2). Gender, age, and income were also included, but only the income was of greater importance among the demographic factors. An increase in income category was associated with increasing odds of more conscious consumer behaviour, with an odds ratio of 1.32, a decrease in the odds of suffering from food insecurity and satisfying needs by 17%, suffering from food stress by 25%, and also decreasing exposure to higher food prices by 43%.

**Figure 2:**
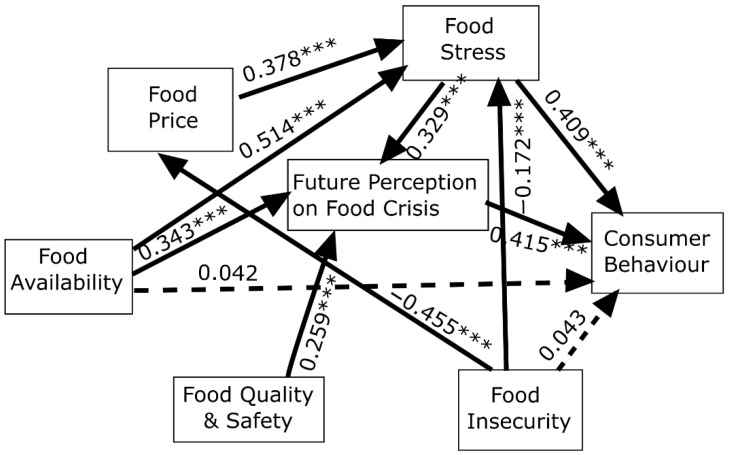
The final path model and path coefficient estimates. Dashed lines indicate the non-significant paths. ***: *p* < 0.001. Source: Authors’ own compilation.

FS1 (food runs out), FA2 (adequate food is not available), FI2 (nutritional needs), producers provide adulterated, and harmful foods (FQS3, FPFC4) all produced an increase in more conscious consumer behaviour. Food stress was increased by FP2 and FA1 and was related strongly to consumer behaviour (CB1-2). Additionally, FQS1 and FI1 increased anxiety. Moreover, food price fluctuations and high prices were associated with food stress (FS1: food runs out) and food insecurity (FI1). FA, FS, and FQS were strongly associated with all the factors of FPFC, and FA and FS had a strong connection. It can also be stated that the CB3 was not of much significance. Based on the findings of the MVORD model, the authors were able to build a conceptual model (see Figure 2).

Figure 2 also provides the parameter estimates in the food security and consumer behaviour model. The PLS-SEM model is an iterative and explanatory technique; therefore, it can identify irrelevant relationships. Statically significant (*p* < 0.05) path coefficients are depicted, and dashed lines indicate no significant paths. It can be seen from Figure 2 that not all hypothesized relationships proved to be significant. For example, the effect of FA and FI was not relevant to consumer behaviour. Table 3 exhibits the proposed hypotheses, confirmed or not confirmed.

The overall model had a good global fit, as the GOF was 0.618, and the quality of the inner and outer model was also excellent, as the R^2^ values were large and the Dillon–Goldstein’s rho exceeded 0.7, and the AVE values were also greater than 0.5, as shown in Table 4. We also applied a bootstrap to validate the parameter estimations in which 500 samples were drawn from the original data proposed by Chin [78]. Then, the mean and standard error of the path coefficients were estimated. Path coefficients considered statistically significant in Figure 2 had low standard errors (at most, 50% of their mean). For the sake of clarity, we consistently report path coefficients with their standard errors and t statistics.

The strongest relationship can be seen between food availability and food stress (B = 0.514; SE = 0.048; t = 10.71; *p* < 0.001) and between food insecurity and food prices (B = 0.455; SE = 0.050; t = 9.10; *p* < 0.001). This means that if food is not available in local shops or adequate food is not available to meet daily demands, consumers will be more anxious. On the other hand, a lower level of food insecurity was strongly connected with higher food prices and fluctuations in prices. However, the increase in food prices caused food stress (B = 0.378; SE = 0.051; t = 7.41; *p* < 0.001), which will have an influence on consumer behaviour in the direction of hoarding, purchasing less food, and reducing waste (B = 0.545; SE = 0.049; t = 11.12; *p* < 0.001). Future perception of food crises was strongly affected by food stress (B = 0.329; SE = 0.051; t = 6.45; *p* < 0.001) and food availability (B = 0.512; SE = 0.049; t = 10.45; *p* < 0.001) and also resulted in a change in consumer behaviour (B = 0.415; SE = 0.047; t = 8.83; *p* < 0.001). Food insecurity and food availability indirectly affected consumer behaviour through food stress and future perceptions of the food crisis.

The matrix’s main diagonal of the numbered columns shows the AVE values, which express the percentage that a given LV explains from its items’ variance. The squared Pearson’s correlation coefficients are given under the main diagonal, while above the main diagonal the significances of the correlation coefficients can be seen. From the AVE values, it is obvious that each LV explained at least an average of 50% of the variance of its items, and the Fornell and Larcker criterion (discriminant validity) was also satisfied as all AVE values for the LVs were higher than the squared intervariable correlations. The highest squared correlations were seen between food stress, future perception of the food crisis, and consumer behaviour. Food availability and food quality and safety had a significant but lower correlation with food insecurity. The three main regressions of the model are food stress (R^2^ = 0.647; SE = 0.043; t = 15.05; *p* < 0.001), future perception and vision (R^2^ = 0.606; SE = 0.041; t = 14.8; *p* < 0.001), and consumer behaviour (R^2^ = 0.591; SE = 0.048; t = 12.32; *p* < 0.001). The proportion of variance explained in the three models by the coefficient of determination (R^2^) was significant.

The major advantage of the PLS-SEM method is that we can differentiate between direct, indirect, and total effects on the outcome variable (consumer behaviour) (Table 5).

Total effects on consumer behaviour can be divided into direct (48.3%) and indirect effects (51.7%). Regarding the direct effects, future perception of the food crisis and food stress are the most influential variables, causing 45.7% and 45% of the direct effects, while food insecurity and food availability contributed only 4.7% and 4.6% to the direct effects. Among indirect effects, food availability had the strongest effect on consumer behaviour (39.9%), while food prices and food insecurity are the second and third most influential factors (19.5% and 17.7%). Analysing the total effects, it can be observed that food stress constituted 29% and food availability 24.7% of the total explained variance in consumer behaviour, and the third factor was future perceptions of food crises, with 22.1%. Food insecurity and food quality and safety had only a small, but significant, effect on consumer behaviour, constituting 7.7% and 5.7% of the explained variance.

Uncertainty in lockdown measures, as well as varying enforcement among regions, restrictions on inter-state mobility, and the lack of transportation capabilities have impacted food supply [21], which is likely to impact food stress, food availability, food prices, food insecurity, and future perceptions of food crises.

Islam et al. in their study found a high prevalence of food stress differs from the results of our studies conducted in Bangladesh, as those studies explain food stress strongly affecting 15.86% of the total variance [41], while our result shows it affecting 29% of the total variance and it being the most influential factor affecting consumer behaviour. Our survey results indicated that 71% of respondents stated their income decreased during COVID-19, and 92% of respondents did not get any aid from any government or private sources during COVID-19. On the other hand, 37% of respondents were eating twice a day during the pandemic, indicating changing eating habits. Similarly, the study by Cariappa et al. found that 92% of respondents’ livelihoods were affected [21].

Similar to other studies in different countries [21,22,91] and in Bangladesh [6], it was found that household income was the root cause of food stress that affects consumer behaviour and food security. Cariappa et al. showed that 75.31% of consumers experienced a price increase across COVID zones [21], while in our study food price was the second most influential factor, at 19.5%, which is comparable with the results of the present study.

A surge in food stockpiling by panicked consumers due to skyrocketing costs and food availability concerns is a crucial factor affecting the food security situation during the lockdown. As a result of the increased COVID-19 crisis, future perceptions of food crises, consumers’ purchasing patterns, and financial situations alter significantly. This study’s findings are consistent with previous research on earlier crises and consumer behaviour. Voinea and Filip [92] discovered that a recession has a significant economic and social influence on consumers by causing them to change their buying behaviour. Moreover, Mansoor and Jalal [93] highlighted changing tendencies in consumer behaviour due to the global financial crisis. The reallocation of luxury and essential consumption, the reduction in income, and the tendency to spend less on higher-priced commodities or to substitute products with other products are all examples of these trends.

## 5. Conclusions and Implications

This study presented significant outcomes to the research community by presenting a new approach to understanding consumer buying behaviour and food choices during pandemics. Since the epidemic outbreak can cause major disruption to production, investment, and consumer expenditures [94,95], this problem is more pronounced in under-developed and developing countries such as Bangladesh. There are rapid changes among consumers in Bangladesh regarding food purchasing, consumption, and perceptions of the food industry.

The findings also point out the vital role of food stress, especially through this crisis. Bangladeshi consumers are more stressed during COVID-19 because most employees are not getting their salary regularly, and thousands of people have lost their jobs. In addition, consumers are more concerned with the future food crisis, price, safety and security, nutrition, and food quality. Furthermore, this study describes the current scenario of consumer psychological stress, food purchase behaviour, food consumption behaviour, and future perception of food in Bangladesh. It also presents the disruption of food production and the food supply chain in Bangladesh.

We found that during COVID-19, producers provide adulterated, harmful foods that increase more conscious consumer behaviour. Food price fluctuations and high prices are associated with food stress because of food scarcity and food insecurity. Food availability, food stress, and food quality and safety issues are strongly associated with all the factors of future perceptions about the food crisis. The effect of food availability and food insecurity was not relevant to consumer behaviour. A strong relationship was evident between food availability and food stress, and between food insecurity and food prices [96]. This indicates that consumers will be more anxious if food is not available in local shops to meet daily demands during COVID-19. Food prices increase food stress, which will influence consumer behaviour in the direction of food hoarding, purchasing less food, and reducing waste [97,98,99].

Moreover, future perceptions of the food crisis are strongly affected by food stress and food availability and will also change consumer behaviour. Food insecurity and food availability indirectly affect consumer behaviour through food stress and future perceptions of the food crisis. Nevertheless, there was a significant correlation between food availability and food quality and safety, but a lower correlation with food insecurity. Regarding only the direct effects, future perceptions of food crises and food stress were the most influential, while food insecurity and food availability contributed to lower levels of direct effects. Among indirect effects, food availability had the strongest effect on consumer behaviour, while food prices and food insecurity were the second and third most influential factors.

The findings of this study meet the aims of the research to explore consumers’ buying behaviour in association with the stress generated from the food supply shortage during the COVID-19 outbreak and the post-outbreak perception of the food industry and food security in Bangladesh. It can be concluded that food stress directly affects consumer behaviour and that both food stress and price seem to influence future food perceptions. On the other hand, food price directly affects food stress, and has a significant relationship with food insecurity, which ultimately affects future food perceptions during COVID-19 outbreaks based on food accessibility, availability, stability, and nutrition.

The research is expected to support governments, producers, stakeholders, and policy planners involved in food production, supply, logistics, and the decision-making process to ensure suitable supply chain management systems during crises, as well as to assist marketers, policymakers, and food consumers during the pandemic.

## 6. Recommendations, Limitations, and Further Research Directions

Almost 71% of respondents in our study reported a drop in income, and households had to change their food waste behaviours during the COVID-19 lockdown. The growing food crisis can be avoided through effective intervention; retail shops need to adapt with new dynamic capabilities to deal with consumers’ needs, demands, and government forces to continue business, and a new agile approach is essential to deal rapidly with the lead time between changes in consumer demand and retailers’ responses. Connecting consumers with local producers is another approach for reducing commodity supply chain disruption. In the context of long-term food security, such practices are strongly recommended for reducing food miles.

COVID-19 had an impact on cross-border transportation between Bangladesh and India. Air and freight constraints, as well as export limits, have exacerbated food supply problems, which must be addressed. Companies could use inventory management to ensure a constant supply of food during storages and lengthier transportations of perishable goods. The government must work together to prevent disruption in the food supply chain and support a wide range of food production and consumption systems, from agriculture to food transport and storage to the capacity of households to afford groceries.

Another suggestion for reducing strain on food production owing to time-related deterioration is for distributors from two different supply chains to share warehouses. When the rate of deterioration increases linearly with time, the shared warehouse technique results in significant cost savings.

During the pandemic, supply chain management should adopt new strategies for organizational change and move away from traditional plans. Retailers should clarify that their priority is to ensure the consumer’s safety and health, and not profit, while delivering the product.

Due to the global epidemic expanding and negatively impacting on economic activities, the World Bank has suggested several initiatives to tackle food security concerns in Bangladesh. Primarily, food safety will have to be observed in particular in order to ensure that: (i) food markets and value chains should function flawlessly both domestically and internationally, (ii) the rural and urban poor, including large informal industries, should retain purchasing power for their social protection, and (iii) input and labour supply must be protected for next-season farm production [100]. Treating food as a “basic commodity” keeps foods flowing and opens specific food, trade, and agricultural input procedures (“green channels”) to ensure supply chains are kept active and working [101] and declaring food processing, marketing, and distribution as necessary services. Everywhere, ensuring that these workers are safe and keeping trade channels open within and across nations ensures the continued functioning of critical aspects of food systems across countries. Finally, it is mandatory to work together in every sector and with every stakeholder to reduce the immediate impacts and redesign all food systems and logistics support during the COVID-19 outbreak crisis to align food production and consumption with sustainable development [102,103,104,105,106].

Although this study aimed to incorporate the potential impacts of FSC disruption on food security and consumer buying behaviour as a result of stringency measures in COVID-19, various other factors, such as the host country’s socio-political status, the level of governance and accountability, the geographic region, the efficiency of human capital, advanced technology, information and communication technology, and FSC infrastructure, can influence purchasing behaviour and the food security status of the country. This is the main limitation of this research. Moreover, due to lockdown, a significant part of the data was collected through an online survey. Therefore, sometimes respondents do not respond with as much empathy to an online survey as they would with a face-to-face survey. Some limitations have to be mentioned with respect to PLS modelling. The interpretation of the model could be more difficult with negative weights, and formative measurements with negative weights could be critical. Multicollinearity could still be an issue in the case of a formative method of modelling. There is no global index available for model validation, and the use of the present goodness-of-fit index is limited. Some variables might have violated the Fornell and Larcker criterion.

Future research should measure consumers’ post-behaviour after the pandemic. Moreover, such studies may investigate the effect of supply chain obstruction from the short-term to the long-term, or how interrupted food supply chains can be reconfigured and sustained, or how new supply chain approaches should be adopted to create food security resilience.

## Figures and Tables

**Table 1:** Descriptive statistics, loadings, and composite reliability of the studied items.

Description	Latent Variable (CR Value)	Manifest Variables **	Mean/Median/Mode	St. Deviation/Interquartile Range	Factor Loading before Exclusion *	Factor Loading after Exclusion *
COVID-19 affects purchasing behaviour.	Consumer Behaviour (0.665)	CB1	4.08/4/5	1.00/1	0.836	0.866
COVID-19 crisis drives me to hoard food.		CB2	3.36/4/4	1.37/2	0.898	0.919
COVID-19 influences me to reduce food waste.		CB3	3.98/4/4	0.99/1	0.569	-
Concerned about running out of food.	Food Stress (0.741)	FS1	3.99/4/5	1.13/1	0.924	0.927
Anxious because of having less money to buy food.		FS2	3.82/4/4	1.06/2	0.834	0.826
Food prices are higher in the crisis.	Food Price (0.675)	FP1	4.30/5/5	0.99/1	0.920	0.922
Cannot afford to buy food because of price hikes.		FP2	3.21/3/2	1.13/2	0.843	0.838
Food is not available in local shops.	Food Availability (0.831)	FA1	3.11/3/4	1.27/2	0.939	0.937
Food amount does not allow me to meet daily demand.		FA2	3.51/4/4	1.23/1	0.904	0.904
Food quality is inferior during COVID-19.	Food Quality and Safety (0.5)	FQS1	3.06/3/3	0.98/2	0.716	0.737
I was feeling unsafe regarding the food supply in the crisis.		FQS2	2.75/3/2	0.97/1	0.549	-
Low quality and adulterated foods.		FQS3	3.15/3/4	1.01/2	0.896	0.947
Feel insecure regarding foods in the crisis.	Food Insecurity (0.76)	FI1	3.91/4/4	0.91/0	0.751	0.721
Not able to meet daily nutritional needs.		FI2	2.38/2/2	1.15/1	0.734	0.770
There will be a big food crisis after the pandemic.	Future Perception of Food Crises (0.699)	FPFC1	3.90/4/4	1.10/2	0.859	0.904
The price of food will be higher after the crisis.		FPFC2	3.87/4/4	0.97/2	0.884	0.886
Food production will increase, and food quality will decrease after the crisis.		FPFC3	2.97/3/4	0.96/2	0.448	-
Producers/suppliers will provide adulterated foods which are harmful to health to create higher food demand		FPFC4	3.50/4/4	0.98/1	0.768	0.795

*, FPFC3 and CB3 should be left out of the final model because of the low factor loadings (<0.5). After the exclusion, most of the loadings were improved. CR Value: Cronbach’s alpha. **, All items were measured on a 1–5 Likert scale, 1: strongly disagree; 2: disagree; 3: neutral; 4: agree; 5: strongly agree. Source: Authors’ own compilation.

**Table 2:** Relative risk measures from the ordered logit analysis.

Factor	Blocks						
	CB	FS	FP	FA	FQS	FI	FPFC
Gender	0.89	1.11	0.39	1.01	1.02	0.68	0.82
Age	1.12	0.68 *	1.53 *	1.23	1.13	1.13	0.91
Income	1.32 ***	0.75 **	0.57 ***	0.93	1.13 **	0.83 *	0.99
CB1	-	1.53 ***	1.19	1.31 *	0.79 **	1.03	1.04
CB 2	-	1.96 ***	0.72 **	1.09	0.90	0.96	1.31 ***
CB 3	-	0.89	0.84	1.16	1.04	0.94	0.83 **
FS1	2.26 ***	-	1.95 ***	0.67 **	0.86 *	0.56 ***	1.31 ***
FS2	1.10	-	1.03	1.56 ***	1.29 **	0.81	1.13
FP1	1.15	0.97	-	1.60 **	1.16 *	1.02	1.11
FP2	0.77 **	1.84 ***	-	1.16	1.16 *	1.01	1.05
FA1	0.94	1.90 ***	1.21	-	1.01	1.05	1.13
FA2	1.42 ***	0.69 *	0.70 **	-	1.38 ***	0.72 *	1.35 ***
FQS1	0.63 ***	1.32 *	0.94	1.177	-	1.02	1.38 ***
FQS2	0.96	0.96	0.82	1.796 ***	-	1.00	0.78 ***
FQS3	1.19 *	1.04	1.43 **	1.011	-	1.08	1.15 *
FI1	0.54 ***	0.74 *	0.43 ***	1.011	1.09	-	0.93
FI2	1.51 ***	1.12	0.92	0.925	0.99	-	0.98
FPFC1	0.96	1.12	1.05	1.390 **	0.74 ***	1.02	-
FPFC2	1.06	1.12	1.21	1.373 *	1.23 **	0.85	-
FPFC3	0.89	1.00	0.90	1.467 **	1.18 **	0.78 *	-
FPFC4	1.37 ***	0.86	1.48 ***	1.299 *	1.18 **	1.24	-
MC Fadden	0.171	0.269	0.208	0.257	0.075	0.086	0.156

*, *p* < 0.05; **, *p* < 0.01; ***, *p* < 0.001. FA, food availability; FS, food stress; FI, food insecurity; FP, food prices; FQS, food quality and safety; FPFC, food perception on food crisis; CB, consumer behaviour. Source: Authors’ own compilation.

**Table 3:** The summary of hypotheses, confirmed or not confirmed.

Variables	(H) No.	Hypotheses	Results
FS	H1a	Food stress has a direct effect on consumer behaviour	Confirmed
	H1b	Food stress has a direct effect on future perceptions of the food crisis	Confirmed
FP	H2	Food price has a direct effect on food stress	Confirmed
FA	H3a	Food availability has a direct effect on consumer behaviour	Not confirmed
	H3b	Food availability has a direct effect on future perceptions of the food crisis.	Confirmed
	H3c	Food availability has a direct effect on food stress	Confirmed
FQS	H4	Food quality and safety have a direct effect on future perceptions of the food crisis.	Confirmed
FI	H5a	Food insecurity has a significant effect on consumer behaviour	Not confirmed
	H5b	Food insecurity has a direct effect on food price	Confirmed
	H5c	Food insecurity has a direct effect on food stress	Confirmed
FPFC	H6	Future perceptions of food crises have a direct effect on consumer behaviour.	Confirmed

Source: Authors’ own compilation

**Table 4:** Quality measures of the inner and outer model and the squared correlations * between latent variables.

Latent Variable	R^2^	DG rho **	1	2	3	4	5	6	7
Food Stress (1)	0.647	0.886	0.771	<0.001	<0.001	<0.001	<0.001	<0.001	<0.001
Food Availability (2)	n.a.	0.922	0.437	0.848	0.004	<0.001	0.002	<0.001	<0.001
Food Price (3)	0.207	0.862	0.390	0.108	0.775	0.023	<0.001	<0.001	<0.001
Food Quality and Safety (4)	n.a.	0.842	0.184	0.265	0.022	0.721	<0.001	<0.001	<0.001
Food Insecurity (5)	n.a.	0.782	0.171 *	0.019 *	0.207 *	0.037 *	0.556	<0.001	<0.001
Future Perception on Food Crisis (6)	0.606	0.872	0.444	0.481	0.189	0.332	0.062 *	0.745	<0.001
Consumer Behaviour (7)	0.591	0.888	0.482	0.352	0.089	0.246	0.055 *	0.497	0.797

*: The square of the negative correlation coefficients were indicated with italics. **: Dillon–Goldstein’s rho; rho stands for the Greek letter and is used to measure correlation. Source: Authors’ own compilation.

**Table 5:** Total, direct, and indirect effects and the ratio of explained variance in social influence.

Relationships	Effects ***		
	Direct	Indirect **	Total *
FA -> FS	0.514	0.000	0.514
FA -> FPFC	0.343	0.169	0.512
FA -> CB	0.042 (4.6%)	0.422 (39.9%)	0.464 (24.7%)
FI -> FP	−0.455	0.000	−0.455
FI -> FS	−0.172	−0.172	−0.344
FI -> FPFC	0.000	−0.113	−0.113
FI -> CB	0.043 (4.7%)	−0.187 * (17.7% *)	−0.144 * (7.7% *)
FQS -> FPFC	0.259	0.000	0.259
FQS -> CB	0.000 (0.0%)	0.107 (10.1%)	0.107 (5.7%)
FP -> FS	0.378	0.000	0.378
FP -> FPFC	0.000	0.124	0.124
FP -> CB	0.000 (0.0%)	0.206 (19.5%)	0.206 (11.0%)
FS -> FPFC	0.329	0.000	0.329
FS -> CB	0.409 (45.0%)	0.136 (12.3%)	0.545 (29.0%)
FPFC-> CB	0.415 (45.7%)	0.000 (0.0%)	0.415 (22.1%)
Total on CB (%)	0.909 (48.3%)	1.058 * (0.972 **) (51.7% **)	1.881 * (100%)

Notes: FA, food availability; FS, food stress; FI, food insecurity; FP, food prices; FQS, food quality and safety; FPFC, food perception on food crisis; CB, consumer behaviour. *, In case of negative effects, the absolute value was used to calculate the total. **, This is the part of the total effect that is indirect. ***, Effect percentages in parenthesis sum up to 100% column-wise. Source: Authors’ own compilation.

## Supplementary Materials

The following are available online at https://www.mdpi.com/article/10.3390/foods10123073/s1, Quesionnaire: Food Security and COVID-19 Crisis from the Consumer Buying Behavior Context—the case of Bangladesh.
