# Supplementary material for: Food Security and the COVID-19 Crisis from a Consumer Buying Behaviour Perspective—The Case of Bangladesh
Source: Foods. 2021 Dec 10;10(12):3073. doi: 10.3390/foods10123073 (PMC8701356; doi:10.3390/foods10123073)
Supplement: Supplementary file 1 [file foods-10-03073-s001.zip › foods-1477774-supplementary.pdf]

Questionnaire

Food Security and COVID-19 Crisis from the Consumer Buying Behavior Context – the case of Bangladesh

Section- A (Demography & General Information About COVID-19)

A. Gender

1. Male 2. Female

B. Age

1. < 20 years 2. 20-30 years 3. 30-40 years 4. > 40 years

C. Occupation

1. Government job holder 2. Private Jobholder 3. Day labor 4. Students 5. Housewife 6. Unemployment 7. Agriculture 8. Business 9. Others/PhD fellow

D. Regular Income (in BDT)

1. 0- 10000 2. 10,000-20,000 3. 20,000-30,000 4. 30000-40000 5. Above 40000

E. The number of people in the household?

1. < 2 persons 2. 3 persons 3. 4 persons 4. > 5 persons

F. Did your current income decrease during the coronavirus (COVID-19) epidemic?

1. Yes 2. No

G. How many times are you eating in a day during COVID 1-9?

1. 2 times 2. 3 times 4. 4 times 5. More than 4 times

H. Are you getting any aid from any sources?

1. Yes 2 No

Section-B: (Measurements construct)

Direction: For each question given below, please tick (√) only one option that best reflects your opinion on the following five point scale:

1	2	3	4	5
Strongly Disagree	Disagree	Neutral	Agree	Strongly Agree

Coding No	Variables (Independent)	Strongly Disagree	Disagree	Neutral	Agree	Strongly Agree
**Consumer Behavior**						
CB1	I think COVID19 affects my purchasing behavior.	1	2	3	4	5

CB2	I think COVID -19 crisis is driving me to food hoarding.	1	2	3	4	5
CB3	I think, COVID-19 influences to reduce the food waste.	1	2	3	4	5
**Food Anxiety**						
FAN1	I'm concerned when my food runs out.	1	2	3	4	5
FAN2	I am so anxious because I have a little money to buy food.	1	2	3	4	5
**Food Price**						
FP1	Food prices are more expensive during COVID-19 than before.	1	2	3	4	5
FP2	I cannot afford the food to buy properly because of the price hike.	1	2	3	4	5
**Food Availability**						
FA1	I think, Food is not available in grocery shops and local shops in my area.	1	2	3	4	5
FA2	I think adequate food is not available to meet the consumer daily demand.	1	2	3	4	5
**Food Quality & Safety**						
FQS1	I think food quality is inferior during COVID-19 than before.	1	2	3	4	5
FQS2	I feel safe regarding food supply from producers during COVID 19?	1	2	3	4	5
FQS3	I think the suppliers provide a low quality of food with adulterated	1	2	3	4	5
**Food Insecurity**						
FI1	During coronavirus (COVID-19), do you feel being secured regarding foods?	1	2	3	4	5
FI2	During coronavirus (COVID-19), are you able to meet your daily nutritional needs?	1	2	3	4	5
**Future Perception about Food Crisis**						
FPFC1	I think, country will face a big food crisis after the pandemic.	1	2	3	4	5
FPFC2	I think, The price of food will be higher after the pandemic.	1	2	3	4	5
FPFC3	I think, after pandemic, Food production will be increased but	1	2	3	4	5

	food quality will decrease					
FPFC4	I think, Producers/ suppliers will provide adulterated foods which are harmful for health to make higher food demand	1	2	3	4	5

"Thanks for your nice Cooperation"
